# Human papillomavirus infection and cervical cancer precursor lesions in women living by Amazon rivers: investigation of relations with markers of oxidative stress

**DOI:** 10.1590/S1679-45082018AO4190

**Published:** 2018-08-09

**Authors:** Bruna Emanuelle Sanches Borges, Elza Baia de Brito, Hellen Thais Fuzii, Cláudia Simone Baltazar, Aline Barreto Sá, Camile Irene Mota da Silva, Gleyce de Fátima Silva Santos, Maria da Conceição Nascimento Pinheiro

**Affiliations:** 1 Programa de Pós-Graduação Em Doenças Tropicais, Universidade Federal do Pará, Belém, PA, Brazil.

**Keywords:** Papillomaviridae, Oxidative stress, Oxidants, Women, Uterine cervical neoplasms, Brazil, Papillomaviridae, Estresse oxidativo, Oxidantes, Mulheres, Neoplasias do colo do útero, Brasil

## Abstract

**Objective:**

To investigate the relation between oxidative stress markers, human papillomavirus infection and cervical cancer precursor lesions.

**Methods:**

The study comprised women aged 14 to 60 years living in communities located by Amazon rivers in the state of Pará (Itaituba, Limoeiro do Ajuru and Bragança, 126, 68 and 43 women respectively). Papanicolau smears and polymerase chain reaction tests for human papillomavirus DNA detection were performed. Blood samples were collected to test malondialdehyde, total and oxidized glutathione levels.

**Results:**

Malondialdehyde, total and oxidized glutathione concentrations did not differ significantly (p>0.05) between women with and without low-grade squamous intraepithelial lesions across communities. Malondialdehyde levels (8.02nmols/mL) were almost five times higher in human papillomavirus-positive compared to human papillomavirus-negative women (1.70nmols/mL) living in Itaituba (statistically significant difference; p<0.05). Malondialdehyde levels did not differ significantly (p>0.05) between human papillomavirus-positive and human papillomavirus-negative women living in remaining communities. Significant (p<0.05) differences in total glutathione levels between human papillomavirus-positive and human papillomavirus-negative women (8.20μg/mL and 1.47μg/mL, respectively) were limited to those living in Bragança.

**Conclusion:**

Malondialdehyde and total glutathione levels were significantly associated with human papillomavirus infection. However, lack of similar associations with squamous lesions suggest oxidative stress alone does not explain correlations with cervical carcinogenesis. Other factors may therefore be involved.

## RESUMO

**Objetivo:**

Avaliar a relação de marcadores de estresse oxidativo com a infecção pelo papilomavírus humano e as lesões precursoras do câncer cervical.

**Métodos:**

Foram incluídas mulheres ribeirinhas da Amazônia (a saber: 126 do município de Itaituba, 68 de Limoeiro do Ajuru e 43 de Bragança), todas do Estado do Pará, de 14 a 60 anos. Foram realizados testes Papanicolau e de reação em cadeia de polimerase para a detecção de DNA do papilomavírus humano. Coleta de amostras de sangue foi realizada para a avaliação do malondialdeído e a determinação da glutationa total e oxidada.

**Resultados:**

As concentrações oxidativas do malondialdeído e os teores de glutationa total e oxidada não apresentaram diferença significativa (p>0,05) nas mulheres com e sem lesão intraepitelial escamosa de baixo grau, em todas as comunidades pesquisadas. Mulheres com papilomavírus humano em Itaituba apresentaram níveis de malondialdeído (8,02nmols/mL) quase cinco vezes maior quando relacionadas àquelas sem o HPV (1,70nmols/mL), com diferença estatística significativa (p<0,05). As outras comunidades não mostraram diferença estatística significativa entre as concentrações de malondialdeído nas mulheres com e sem papilomavírus humano (p>0,05). Os teores de glutationa total mostraram diferença significativa (p<0,05) somente em Bragança naquelas com (8,20µg/mL) e sem a infecção pelo papilomavírus humano (1,47µg/mL).

**Conclusão:**

Houve associação significativa da resposta oxidativa do malondialdeído e da glutationa total com a infecção pelo papilomavírus humano, porém não houve diferença quando associada à lesão escamosa, sugerindo que o estresse oxidativo isoladamente não explica a relação com a carcinogênese do colo uterino, que deve ser influenciada ainda por outros fatores.

## INTRODUCTION

Cervical cancer is a curable disease when diagnosed early; still it is associated with high morbidity and mortality rates in developing countries.^1^ Cervical carcinogenesis depends upon some human papillomavirus (HPV)-associated factors, such as HPV type, immune system and genetics.^2^ Oxidative stress is responsible for the production of reactive oxygen species (ROS) and is thought to play an important role in this process. Reactive oxygen species cause irreversible damage to important biomolecules, such as membrane lipids, proteins and DNA, contributing to cell injury and death. This dysfunction may impact the development of cervical cancer precursor lesions as well as HPV persistence.^3^

Two cooperative mechanisms are thought to occur between oxidative stress and HPV: (1) oxidative stress genotoxic activity and HPV-induced genomic instability acting independently to generate molecular damage required to trigger the development of neoplastic cells; and (2) oxidative stress interaction with one or more stages of neoplastic initiation and/or progression induced by HPV infection.^2^

Malondialdehyde (MDA), a product of cell membrane peroxidation, is thought to be a major marker of cancer severity. Higher MDA levels have been reported in patients with advanced-stage compared to early-stage cancer.^4^ Malondialdehyde is also thought to be a potential global plasma biomarker of oxidative damage.^5^

Other markers, such as glutathione and enzymes in the MDA catalytic cycle, have been associated with antioxidant defense changes and increased oxidative stress, both of which are thought to be carcinogenic factors. Hence, quantitative determination of glutathione levels may reveal potential correlations between reduced levels of antioxidant enzymes, such as glutathione peroxidase (GSH-Px), and increased levels of DNA base damaged in response to oxidative stress.^6^

Lower GSH-Px activity, lower plasma antioxidant vitamin levels and higher MDA concentrations were documented in women suffering from cervical intraepithelial neoplasia or invasive cervical cancer compared to women in the Control Group.^7^ Higher MDA levels were also reported in cervical cancer patients compared to controls,^8^ along with lower levels of antioxidant enzymes superoxide dismutase (SOD), catalase (CAT) and GSH-Px.

Studies investigating associations between cervical cancer precursor lesions, HPV infection and oxidative stress in specific populations may bring significant contributions to the battle against cervical cancer. People living along river banks rely on traditional fishing for subsistence and commercial reasons, and are geographically widely dispersed and distributed. These populations are also prey to socio-economic constraints to education, housing, diet and health. Such living conditions are thought to play a significant role in high cervical cancer rates in Northern Brazil, where communities living by Amazon rivers are many, and poorly investigated.

## OBJECTIVE

To investigate oxidative and antioxidative responses associated with HPV infection and cervical cancer precursor lesions in an effort to determine the role of oxidative stress in cervical carcinogenesis in women with particular sociocultural features.

## METHODS

An observational, cross-sectional study carried out between 2013 and 2014. The study sample comprised sexually active women aged 14 to 60 years, living by Amazon rivers, and enrolled in the *Programa Nacional de Controle do Câncer do Colo do* Útero (PNCCCU) [National Program to Control Cervical Cancer]. Other inclusion criteria were permanent resident status and visit to the local primary health care unit for Papanicolau test. Women who were unable to provide information required for the survey were excluded. All participants signed an informed consent term. The non-probabilistic sample comprised 237 women living in different municipalities in the state of Pará, as follows: 126 women living in two communities in Itaituba, 68 women living in two communities in Limoeiro do Ajuru, and 43 women living in one community in Bragança.

Cervicovaginal samples for Papanicolau smear and polymerase chain reaction (PCR) detection of HPV DNA were collected by physicians involved in the research project, using endocervical brush and Ayre spatula. Papanicolau smears were analyzed by a research team cytopathologist and results disclosed on the spot. HPV DNA investigation was performed by expert professionals according to well-established methods.^9^

Blood samples were obtained for oxidative stress tests. MDA levels were measured as thiobarbituric acid reactive substances (TBARS) in cell membranes, according to standard methods.^10^ Whole venous blood samples (3mL) were collected for total (total GSH) and oxidized (GSSG) glutathione determination. Samples were deproteinized using 10% trichloroacetic acid (TCA) and ether, then analyzed according to well-established methods after complete ether evaporation.^11^

Results were presented in tables. Quantitative variables were analyzed using descriptive statistics expressed as median, first and third quartiles. The non-parametric Mann-Whitney test was used to compare oxidative stress levels between women with and without low-grade squamous intraepithelial lesions (LSIL) and between HPV-positive and HPV-negative women. The level of significance was set at 5% (p<0.05). Variables were entered into Excel spreadsheets (Excel software version 2010) to build a database, and then analyzed using BioEstat 5.0.

This study was carried out in compliance with ethical standards determined by resolution 466/12 of *Conselho Nacional de Saúde* (CNS) [National Health Council] of the Ministry of Health, and approved by the Research Ethics Committee, opinion no. 334.524, in July 2013, CAAE: 18447613.8.0000.5172.

## RESULTS

Malondialdehyde levels did not differ significantly between women with and without LSIL in investigated communities (p>0.05) (Table 1). Worthy of notice, MDA levels in affected women living in Itaituba (4.52nmols/mL) were much higher compared to women living in Limoeiro do Ajuru (0.23nmols/mL), and three times higher than in those living in Bragança (1.42nmols/mL).

**Table 1:** Malondialdehyde levels, in women with and without cervical cancer precursor lesions

Low-grade squamous intraepithelial lesion	Itaituba	p value*	Limoeiro do Ajuru	p value*	Bragança	p value*
	median; Q1-Q3		median; Q1-Q3		median; Q1-Q3	
With lesion	(n=11)	0.120	(n=3)	-	(n=4)	0.116
	4.52; 1.74-7.98		0.23; 0.21-0.26		1.42; 1.20-1.82	
Without lesion	(n=115)		(n=65)		(n=39)	
	1.71; 0.86-5.87		0.54; 0.28-1.40		2.50; 1.46-3.60	

* p<0.05 statistically difference (Mann-Whitney test).

Q1: first quartile; Q3: third quartile.

Total GSH and GSSG levels did not differ significantly between women with and without LSIL in this sample (p>0.05) (Table 2). However, higher total GSH and GSSG levels were detected in women with LSIL compared to unaffected women in Limoeiro do Ajuru and Bragança, but not in Itaituba.

**Table 2:** Total glutathione and oxidized glutathione levels, in µg/mL, in women with and without cervical cancer precursor

Low-grade squamous intraepithelial lesion	Itaituba	p value*	Limoeiro do Ajuru	p value*	Bragança	p value*
	median; Q1-Q3		median; Q1-Q3		median; Q1-Q3	
Total GSH						
With lesion	(n=11)	0.869	(n=3)	-	(n=4)	0.403
	2.03; 1.35-2.08		5.42; 4.12-5.91		2.97; 2.51-3.72	
Without lesion	(n=115)		(n=65)		(n=39)	
	1.81; 1.43-2.30		3.80; 2.95-6.80		1.60; 1.08-4.42	
GSSG						
With lesion	1.39; 1.36-2.49	0.729	2.71; 1.00-4.38	-	1.55; 1.30-1.90	0.464
Without lesion	1.53; 1.23-2.21		2.20; 1.72-2.92		1.30; 0.91-1.76	

* p<0.05 statistically difference (Mann-Whitney test).

Q1: first quartile; Q3: third quartile; Total GSH: total glutathione; GSSG: oxidized glutathione.

HPV-positive women living in Itaituba had significantly (p<0.05) higher MDA levels compared to HPV-negative women (8.02nmols/mL and 1.70nmols/mL, respectively, *i.e*., almost five-fold increase) (Table 3). Malondialdehyde levels did not differ significantly (p>0.05) between HPV-positive and HPV-negative women living in remaining communities.

**Table 3:** Malondialdehyde levels, in human papillomavirus positive and negative women

HPV	Itaituba	p value*	Limoeiro do Ajuru	p value*	Bragança	p value*
	median; Q1-Q3		median; Q1-Q3		median; Q1-Q3	
HPV positive	(n=17)	0.003	(n=8)	0.313	(n=5)	0.161
	8.02; 1.54-8.05		0.54; 0.13-0.88		3.90; 2.50-4.70	
HPV negative	(n=109)		(n=60)		(n=38)	
	1.70; 0.85-4.41		0.51; 0.27–1.40		2.42; 1.40-3.60	

* p<0.05 statistically difference (Mann-Whitney test).

Q1: first quartile; Q3: third quartile; HPV: human papillomavirus.

Total GSH levels differed significantly (p<0.05) between HPV-positive (8.20µg/mL) and HPV-negative (1.47µg/mL) women living in Bragança only (Table 4). In contrast, total GHH and GSSG levels were higher in HPV-positive compared to HPV-negative women living in Limoeiro do Ajuru and Bragança, although not in Itaituba.

**Table 4:** Total glutathione and oxidized glutathione levels, in mg/mL, in human papillomavirus positive and negative women

Total GSH	Itaituba	p value*	Limoeiro do Ajuru	p value*	Bragança	p value*
	median; Q1-Q3		median; Q1-Q3		median; Q1-Q3	
HPV						
Positive	(n=17)	0.650	(n=8)	0.789	(n=5)	0.021
	1.95; 1.43-2.32		3.80; 3.37-5.52		8.20; 3.20-8.60	
Negative	(n=109)		(n=60)		(n=38)	
	1.80; 1.43-2.25		3.77; 2.83-6.50		1.47; 1.04-4.07	
GSSG						
HPV positive	1.43; 1.36-1.87	0.599	4.10; 2.80-6.35	0.076	1.40; 1.21-1.60	0.690
HPV negative	1.55; 1.29-2.29		1.86; 0.99-3.46		1.30; 0.85-1.78	

* p<0.05 statistically difference (Mann-Whitney test).

Q1: first quartile; Q3: third quartile; HPV: human papillomavirus; GSH: total glutathione; GSSG: oxidized glutathione.

High-grade squamous intraepithelial lesions (HSIL) were not detected in women in this sample.

## DISCUSSION

The relation between oxidative stress and cervical cancer has been extensively investigated.^2,3,7,8^ Still, studies examining similar relations in populations living along Amazon rivers are lacking.

Higher MDA levels in women with LSIL and HPV-positive women living in Itaituba compared to those living in remaining communities investigated may reflect exposure to hazardous substances, such as mercury, given the intense local mining activity. High levels of mercury exposure have been reported in this population due to consumption of fish contaminated with methylmercury. Methylmercury is poisonous to humans and has been associated with neurological changes,^12^ genotoxicity^13^and increased oxidative stress.^14^

However, MDA concentrations were measured using the TBARS assay. Although a widely used, inexpensive and user-friendly test, this assay is non-specific and may be impacted by several substances, such as sugars, amino acids and bilirubin. Malondialdehyde is thought to be an important marker of oxidative stress-induced cell membrane lipid peroxidation.^4,15^

In Limoeiro do Ajuru and Bragança, MDA levels were lower in women with LSIL compared to unaffected women. Contrasting evidence indicating malignant neoplasms are able to release free radicals into the bloodstream have been given elsewhere.^8,16^ In Itaituba, higher levels were documented in women with LSIL. However, there were no significant differences between affected and non-affected women in these communities.

According to Valentini et al.,^17^ blood concentrations of essential metals, carotenoids, vitamin E and some other nutrients fluctuate over the course of a year due to eating frequency and food availability, with potential impacts on oxidative stress. Community location, sex, smoking and eating habits may also interfere with micronutrient levels in the human body.

Importantly, “reference ranges given for MDA levels are highly variable and may reflect different experimental conditions between trials”.^18^

Also, differences in measurement units between this and previous studies made comparisons between oxidative and antioxidative marker levels difficult.

Gonçalves et al.,^19^investigated associations between oxidative stress and cervical cancer progression and revealed two to three-fold increases in TBARS levels in erythrocytes of patients with LSIL, HSIL or cervical cancer. Likewise, in Itaituba, MDA levels were almost three times higher in women with LSIL compared to unaffected women. Higher mean MDA levels were reported by Nirmala et al.,^20^ in cervical cancer patients compared to unaffected patients.

Higher MDA levels were also reported in patients suffering from different types of cancer, including oropharyngeal and colorectal cancer, compared to healthy patients.^4,21^

Higher MDA levels were documented in HPV-positive women in all communities investigated in this study. However, significant differences were limited to women living in Itaituba. This may have reflected the larger sample size in this region. Higher MDA levels may have reflected HPV-induced ROS imbalances and resulting oxidative stress. Epidemiological, clinical and biochemical evidence suggesting that ROS may potentiate viral infection and infection persistence have also been given.^2^

Similar to this study, significantly higher serum MDA levels in women with HPV-related genital warts have been documented by Cokluk et al.^22^ However, findings in this study were limited to HPV positivity (*i.e*., did not involve genital warts). Williams et al.,^23^ observed viral E6 oncogene expression was associated with elevated ROS levels, both in HPV-positive and HPV-negative cells, and increased host cell DNA damage. Elevated ROS levels were thought to be associated with reduced antioxidant defenses, as shown by decreased SOD and GSH-Px levels in response to E6 expression. However, that study did not include any of the oxidative stress markers used in this analysis.

In Itaituba, women affected with LSIL had higher total GSH and lower of GSSG (oxidized form of glutathione) levels compared to unaffected women; this may be associated with antioxidant defense mechanisms such as reduced glutathione (GSH) activation to in response to the neoplastic condition. Just as GSSG, which is thought to be the most powerful antioxidant in the body, GSH is part of the total glutathione pool and may be regulated by diet.^24,25^ Fish is a major dietary component among people living in this Amazon region, and may contain minerals with antioxidative potential, such as selenium.^17^Selenium may mitigate toxic metal (and mercury) toxicity via oxidative and antioxidative mechanisms, thereby contributing to cell defense against free radicals.^26^

Valentini et al.,^17^ demonstrated higher selenium levels in populations living by the Tapajós river, such as the Itaituba community. In that study, normal or above normal selenium levels were detected in roughly 67.1% and 31.7% of participants, respectively.

Likewise, higher levels of antioxidant enzymes CAT and GSH were reported by Maldonado et al.,^16^ in women with HSIL who had not started treatment compared to those who had.

Higher total GSH and GSSG levels, documented in women with LSIL compared to unaffected women living in Limoeiro do Ajuru and Bragança, seem to express oxidative responses, given the elevated GSSG levels. GSSG, a product of GSH oxidation mediated by the GSH-Px enzyme, is a toxic manifestation of oxidative stress. Oxidized glutathione is an important indicator of cellular function and may be altered in several pathological conditions.^27^

Higher total GSH and GSSG levels in women living in Limoeiro do Ajuru and Bragança support previous findings of increased circulating levels of oxidative substances in patients with malignant neoplasia,^4,8,16,21^ even though only precursor or pre-malignant lesions were diagnosed in women in this sample.

Higher total GSH and lower GSSG levels were observed in HPV-positive compared to non-infected women in Itaituba. Just as in other women with LSIL living in the same region, this may have reflected antioxidant activity against the HPV virus. Higher levels of total GSH were also documented in HPV-positive compared to HPV-negative women living in Limoeiro do Ajuru and Bragança, with statistically significant differences between those living in Bragança. Total GSH may reflect oxidative responses, given the higher GSSG levels. This may be explained by HPV-induced oxidative stress.^2^ Also, oxidative stress responses are common in the inflammatory phase of viral infections due to ROS production by neutrophils and macrophages.^23^

Findings of this study are corroborated by previous reports Boisio et al.,^28^ of lower GSH and higher GSSG levels in HPV-positive patients. Similar results have been published by Kwaśniewska et al.,^29^ in that study, lower plasma GSH and higher plasma GSSG levels were documented in HPV-positive women and women with cervical dysplasia and cervical cancer.

Sample size was the major limitation in this study and reflects the geographical isolation of the investigated communities, which are mostly reached by river transportation. Access to these communities is even more limited in the first few months of the year due to frequent episodes of heavy rainfall. Local access to health care facilities is limited for the same reason.

Findings of this study may support future investigations of factors associated with oxidative stress markers and the role of oxidants, antioxidants and other elements in cervical carcinogenesis and HPV infection in vulnerable populations, such as those living along rivers.

## CONCLUSION

This study revealed significant relations between oxidative responses associated with malondialdehyde and total glutathione activity and human papillomavirus infection. However, lack of associations with squamous lesions suggest oxidative stress *per se* does not explain the relation with cervical carcinogenesis and indicates that other factors are potentially involved.
